# Frequency domain broadband short-wave infrared spectroscopy for measurement of tissue optical properties from 685 to 1300 nm

**DOI:** 10.1117/1.JBO.30.4.045001

**Published:** 2025-04-25

**Authors:** Diana Suciu, Thao Pham, Lina Lin Wei, Shripreetika Guruprasad, Darren Roblyer

**Affiliations:** a Boston University, Department of Biomedical Engineering, Boston, Massachusetts, United States; b Boston University, Department of Electrical and Computer Engineering, Boston, Massachusetts, United States

**Keywords:** diffuse optics, shortwave infrared light, water, lipids, absorption spectra

## Abstract

**Significance:**

Extending frequency domain diffuse optical spectroscopy (FD-DOS) into the short-wave infrared (SWIR) region has the potential to improve measurements of key biological tissue chromophores such as water and lipids, given their higher absorption in SWIR compared with near-infrared wavelengths. Few studies have explored FD-DOS in the SWIR range.

**Aim:**

We present the first demonstration of a frequency domain broadband SWIR spectroscopy (FD-Bb-SWIRS) system to measure optical properties from 685 to 1300 nm.

**Approach:**

A custom hybrid system was developed, combining discrete frequency domain measurements from 685 to 980 nm with broadband continuous wave measurements from 900 to 1300 nm. This setup provided absolute absorption ($\mu_{a}$) spectra from 685 to 1300 nm. Validation was performed using mineral oil-based solid phantoms, deuterium oxide ($D_{2}O$) liquid phantoms, and desiccating porcine tissue.

**Results:**

The FD-Bb-SWIRS system was sensitive to changes in $\mu_{a}$ from varying concentrations of absorbers in solid and liquid phantoms. *Ex vivo* measurements of $\mu_{a}$ spectra indicated differences in tissue water content across different porcine tissue samples during baseline and desiccation.

**Conclusions:**

FD-Bb-SWIRS is highly sensitive to $\mu_{a}$ in the 685 to 1300 nm range and enables precise quantification of water in biological tissues. It represents a significant step forward in advancing SWIR-based optical spectroscopy for clinical applications.

## 1 Introduction

Diffuse optical spectroscopy (DOS) is a technique that leverages light propagation in highly scattering media to measure tissue optical properties. This technique is also commonly referred to as near-infrared spectroscopy (NIRS) when utilizing wavelengths in the near-infrared (NIR) region (∼650 to 1000 nm). DOS and NIRS have been widely applied in medical diagnostics in applications ranging from cancer to neuroscience.^1^ Three types of NIRS modalities are commonly used in biological measurements: continuous wave (CW), frequency domain (FD), and time domain (TD).^1^^–^^3^ Among these techniques, only FD-NIRS and TD-NIRS allow for the decoupling of absolute absorption ($\mu_{a}$) and reduced scattering ($\mu_{s}^{\prime }$) from the measured optical signal.^4^ Specifically, for FD-NIRS, the absolute measurements of both $\mu_{a}$ and $\mu_{s}^{\prime }$ can be achieved using calibrated amplitude and phase information of the detected light after traveling through tissue.^1^^,^^5^ One of the limitations of FD-NIRS is the added instrumental complexity compared with CW-NIRS, which typically restricts absolute measurements of optical properties to only a few wavelengths simultaneously. To address this, hybrid FD + CW systems have been developed to acquire absolute broadband $\mu_{a}$ and $\mu_{s}^{\prime }$ spectra.^5^^–^^7^ Though prior NIRS techniques have focused on quantifying oxygenated and deoxygenated hemoglobin (${\mathrm{HbO}}_{2}$ and Hb, respectively),^1^^,^^2^ there has been a growing interest over the last decade in using diffuse optical techniques to measure tissue concentrations of water and lipids.^8^^–^^11^

**Fig. 1:**
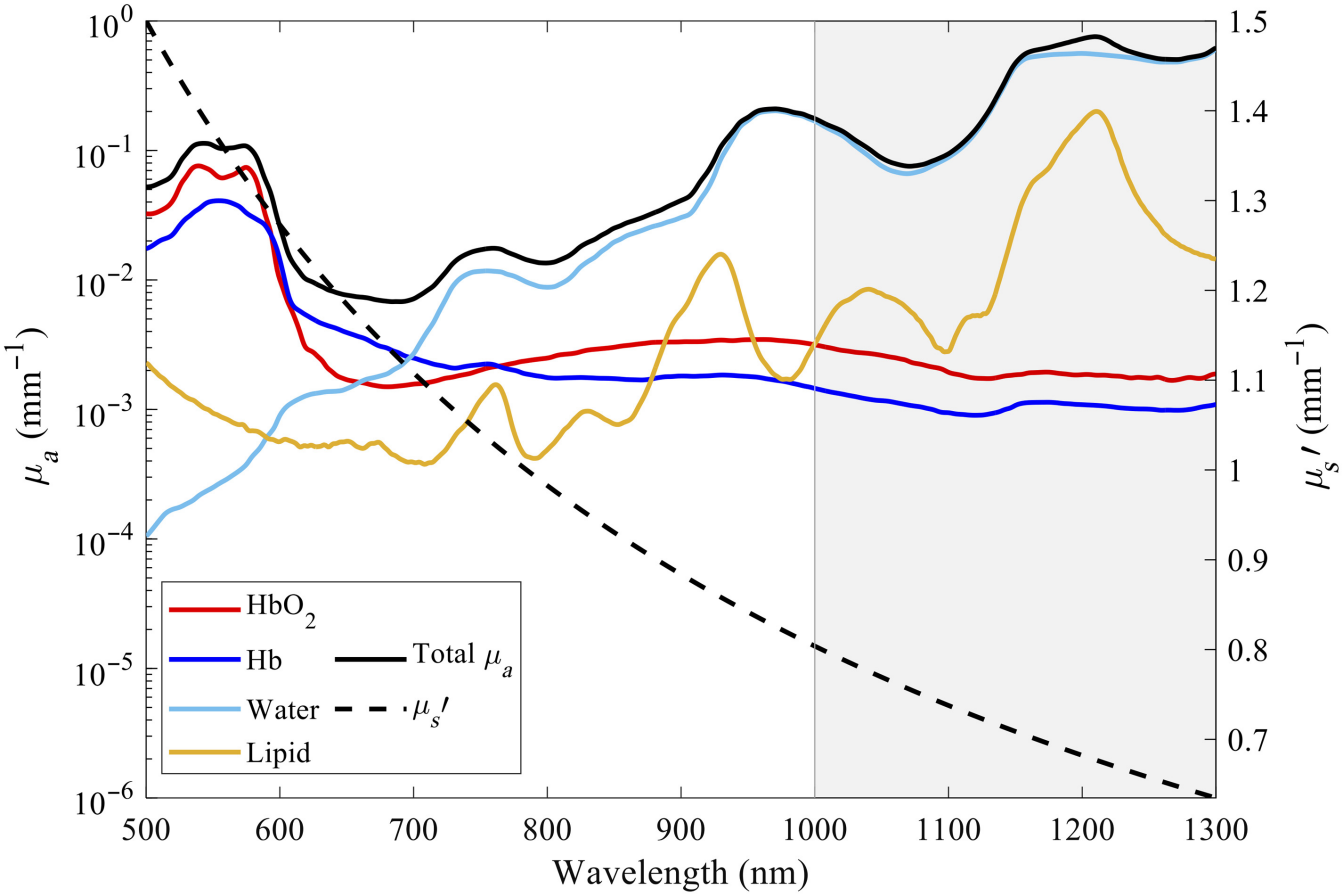
Spectra of absorption coefficients ($\mu_{a}$) and the resulting reduced scattering coefficient ($\mu_{s}^{\prime }$) for four dominant chromophores (oxyhemoglobin ${\mathrm{HbO}}_{2}$, deoxyhemoglobin Hb, water, and lipids) of human tissue. The total $\mu_{a}$ is calculated by adding the individual chromophore absorptions to get the human tissue absorption spectra. The region with white background indicates the NIR spectrum (∼650 to 1000 nm). The shaded region indicates the SWIR spectrum (1000 nm and longer). These plots are generated from values obtained from typical muscle tissue chromophore concentrations, such as total hemoglobin ([HbT])=117  μM, oxygen saturation $({\mathrm{StO}}_{2})=65\%$, [water] = 60%, [lipid] = 20%), $\mu_{s}^{\prime }\;(500\;\mathrm{nm})=1.5\;{\mathrm{mm}}^{-1}$, and scattering power b=0.9.^23^^–^^32^ The sources of extinction coefficients used to generate this figure are listed in the Appendix.

Noninvasive optical measurements of water and lipids may provide utility in a wide range of clinical applications, including chronic conditions such as end-stage kidney disease and heart failure, and monitoring the side effects of cancer treatments among others.^12^^–^^17^ Other applications include hydration monitoring of athletes and a more accurate assessment of body composition during intentional weight loss or gain.^18^^,^^19^ To date, however, most optical techniques designed to monitor water and lipids in biologic tissues have focused on using NIR wavelengths, especially the 900- to 1000-nm range.^20^^–^^22^ Compared with NIR, the short-wave infrared (SWIR) wavelength range (often defined as 1000 to 2000 nm) potentially enables more accurate measurements of water and lipids, as these chromophores exhibit strong absorption in this region, with minimal interference from ${\mathrm{HbO}}_{2}$ and Hb (as seen in Fig. 1). The SWIR wavelength range may also provide deep tissue measurements in certain circumstances, due to the lower tissue scattering and lower melanin absorption compared with NIR wavelengths.^11^ There are a handful of prior studies that have explored SWIR wavelengths for monitoring water and lipids using diffuse optical technologies.^9^^,^^10^^,^^23^ To our knowledge, there has been no prior work developing SWIR in a combined FD and CW system or similar technology to provide absolute tissue optical properties.

In this study, we developed a hybrid FD and CW system to measure broadband and absolute $\mu_{a}$ and $\mu_{s}^{\prime }$ up to 1300 nm. This frequency domain broadband SWIR spectroscopy (FD-Bb-SWIRS) system utilizes FD-NIRS measurements from 685 to 980 nm and broadband CW measurements from 900 to 1300 nm. Using broadband $\mu_{a}$, we demonstrated that water and lipid concentrations can be obtained in phantoms and porcine tissue samples. Compared with other current SWIR techniques such as CW-SWIRS and spatial frequency domain imaging (SWIR-SFDI),^10^^,^^11^^,^^33^^–^^35^ our approach offers several advantages, including the use of single source-detector separation (SDS), direct extraction of optical properties, and greater sensitivity to deeper tissue regions.

## 2 Materials and Methods

### 2.1 Wavelength Selection and Inverse Model Considerations

One of the challenges of using FD technology with SWIR wavelengths is the combination of high $\mu_{a}$ and low $\mu_{s}^{\prime }$ typically found in biological tissue. This combination of optical properties typically renders the diffusion equation (i.e., the analytical solution to the P1 diffusion approximation of the Boltzmann transport equation with semi-infinite boundary conditions) inaccurate.^3^^,^^7^ To address this issue, we evaluated the feasibility of using FD measurements in the SWIR range through the Monte Carlo look-up table (MC-LUT) model (detailed in Sec. 2.3), which are accurate beyond the diffusive regime.^4^

We first conducted a simulation study to estimate the uncertainties in optical properties based on the uncertainties in measured FD diffuse reflectance across a wide range of tissue optical properties, spanning those anticipated in the NIR and SWIR wavelength bands. We explored the range of $\mu_{a}$ from 0.002 to $0.2\;{\mathrm{mm}}^{-1}$ and a range of $\mu_{s}^{\prime }$ from 0.2 to $2\;{\mathrm{mm}}^{-1}$. For each combination of $\mu_{a}$ and $\mu_{s}^{\prime }$, we calculated FD reflectance using the MC-LUT forward model with a modulation frequency of 150 MHz. Then, for each FD reflectance value generated, we created a set of 1000 simulated FD data by introducing Gaussian random noise to the ground truth value. We added zero-mean Gaussian noise with σ=1% to the amplitude and similarly zero-mean Gaussian noise with σ=1  deg to the phase of the reflectance data. Throughout this simulation, we assumed that the noise was consistent across all optical property values. Subsequently, optical properties were recovered from the simulated data by applying the MC-LUT inverse model. The uncertainties in $\mu_{a}$ and $\mu_{s}^{\prime }$ associated with the uncertainties in reflectance data were estimated as standard deviations of the recovered optical property distributions for each specific set of ground truth optical property values.

### 2.2 Instrumentation

**Fig. 2:**
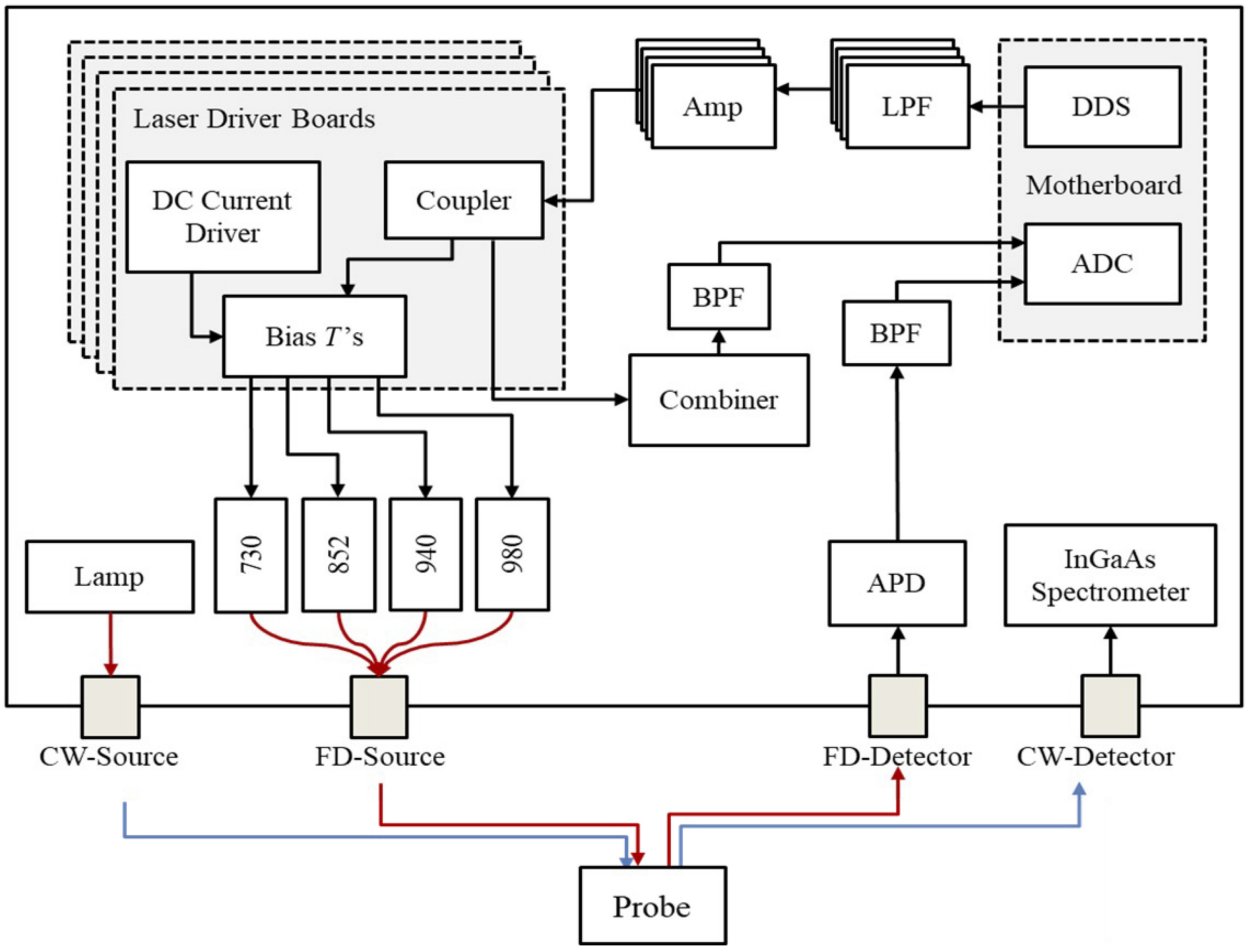
Schematic diagram of the custom FD-Bb-SWIRS system. Key components include the following: ADC, analog-to-digital converter; APD, avalanche photodiode; DC, direct current; DDS, direct digital synthesizer; LPF, low-pass filter; Amp, amplifier; BPF, bandpass filter. FD sources (730, 830, 940, and 980 nm) and FD detector are indicated by red lines, and CW source and detector are indicated by blue lines.

The FD-Bb-SWIRS system combined an FD-NIRS component and a broadband CW-SWIRS component (Fig. 2). Details of the custom digital FD-NIRS component have been described elsewhere.^36^ Depending on the specific experiment, the FD system used illumination wavelengths of either 730, 852, 940, and 980 nm (LP730-SF15 Thorlabs, LP852-SF30 Thorlabs, and LP940-SF30 Thorlabs, and LDT-980-100-MM CNI) or 685, 730, 852, 915, and 980 nm (FMXL685-025SFOB BlueSky, LP730-SF15 Thorlabs, LP852-SF30 Thorlabs, LP915-SF40 Thorlabs, and FMXL980-025SFOB BlueSky). The laser illumination power for both systems ranged from 6 to 14 mW per laser depending on the wavelengths, such that each system’s combined laser power output was within the American National Standards Institute safety standard. Lasers were modulated at discrete frequencies between 127 and 177 MHz. A set of direct digital synthesizer (DDS) boards was used to simultaneously modulate each laser diode at the selected modulation frequency. A broad-spectrum tungsten halogen lamp (HL-2000-HP-B, Ocean Optics, Orlando, Florida, United States; wavelength range from 360 to 2400 nm) was used as the illumination source for the CW measurements.

**Fig. 3:**
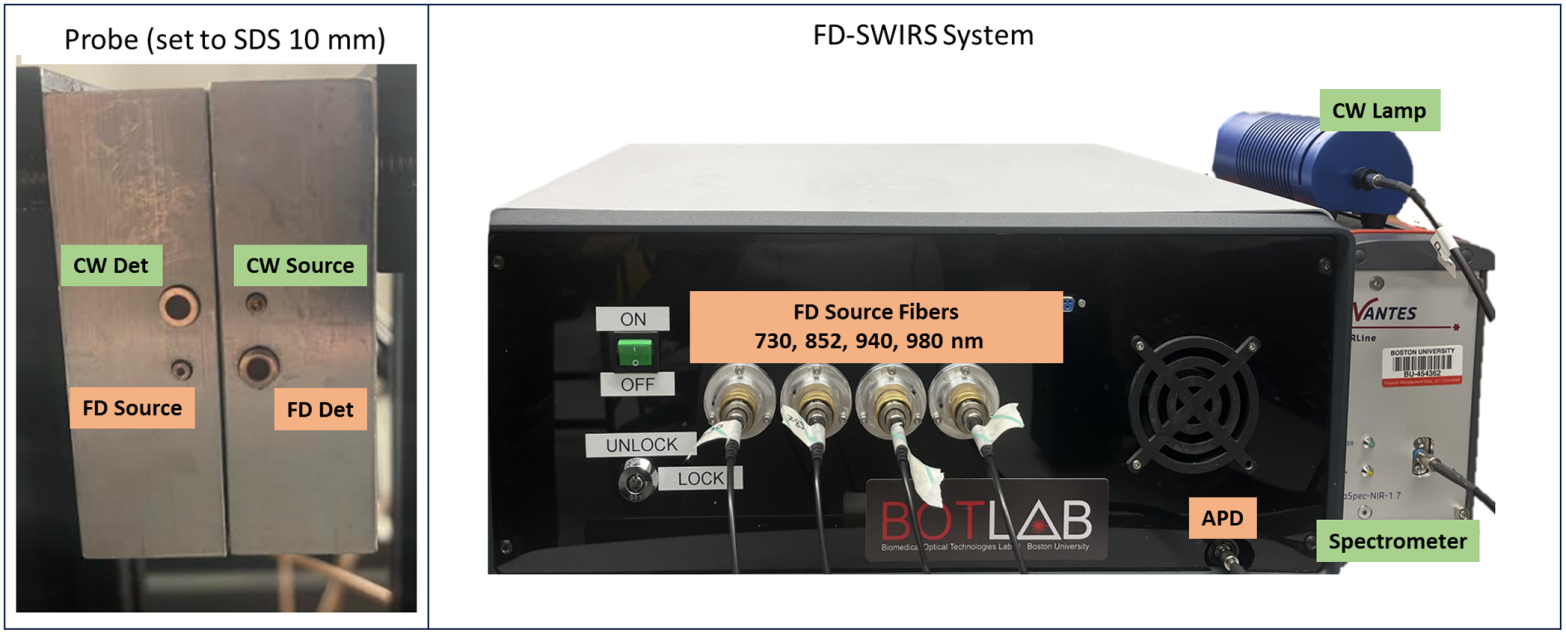
(a) Adjustable probe containing the FD and CW source and detector fibers set to an SDS of 10 mm. (b) FD-Bb-SWIRS system. The black enclosure houses the electronic system which is controlled by a laptop.

Custom optical fibers were used for both FD and CW measurements (Fiberoptic Systems, Simi Valley, California, United States). An eight-in-one source fiber was used for FD measurements in which eight 400-μm core diameter fiber bundles were combined into a single straight ferrule to deliver light to the tissue sample. A single 3-mm active area fiber was used to deliver CW broadband light to the sample. A 2.3-mm fiber (NA: 0.55) was used for FD detection, and a 1-mm fiber (NA: 0.55) was used for CW detection. All four fibers were housed in a custom aluminum probe holder which was fabricated to position fibers on the sample as seen in Fig. 3.

For FD-NIRS detection, a 3-mm-diameter silicon avalanche photodiode (APD) (S11519, Hamamatsu, Shizuoka, Japan) was used to detect laser light. A 250-mega-sample per second analog-to-digital converter (ADC) was used to digitize the APD signal. The Goertzel algorithm was implemented in the system microprocessor to calculate the phase and amplitude of the FD reflectance measurements. The CW-SWIRS remitted broadband light was detected using a 256-pixel uncooled InGaAs spectrometer (AvaSpec-NIR256-1.7-EVO, Avantes, Apeldoorn, the Netherlands). The grating and slit width were selected to achieve a usable measurement range of 900 to 1300 nm with a spectral resolution of 22 nm full width at half maximum. The exposure time of the broadband light source varied depending on the experiment and was adjusted to maximize photon counts while avoiding spectrometer saturation. To ensure reliable measurements, we also measured a dark phantom (fabricated with a high nigrosin concentration as an absorbing agent) using exposure times similar to those used for sample measurements. This allowed us to confirm that the sample intensity counts stayed well above the dark phantom measurements (dark noise) at all wavelengths. The wavelengths at 915, 940, and 980 nm used by the FD-NIRS components of the systems overlapped with the spectral range of the spectrometer.

The FD-Bb-SWIRS system was controlled by a custom graphic user interface written in MATLAB (MathWorks Inc., Natick, Massachusetts, United States) and operated on a Windows 10 laptop. The FD and CW data were collected sequentially, with the FD amplitude and phase data acquired first at an acquisition time of ∼11  ms per measurement, followed by the CW reflectance data collection at 25 to 1200 ms per measurement (experiment dependent). During FD data collection, the CW broad-spectrum lamp was turned off, and only the FD lasers were active. Conversely, during CW data collection, the FD lasers were switched off and the broad-spectrum lamp was turned on. Given the total acquisition time of 35 to 1211 ms, we do not anticipate significant optical, thermodynamic, or other physiologic changes that would impact the system’s ability to capture static tissue optical properties.

### 2.3 Data Processing

**Fig. 4:**
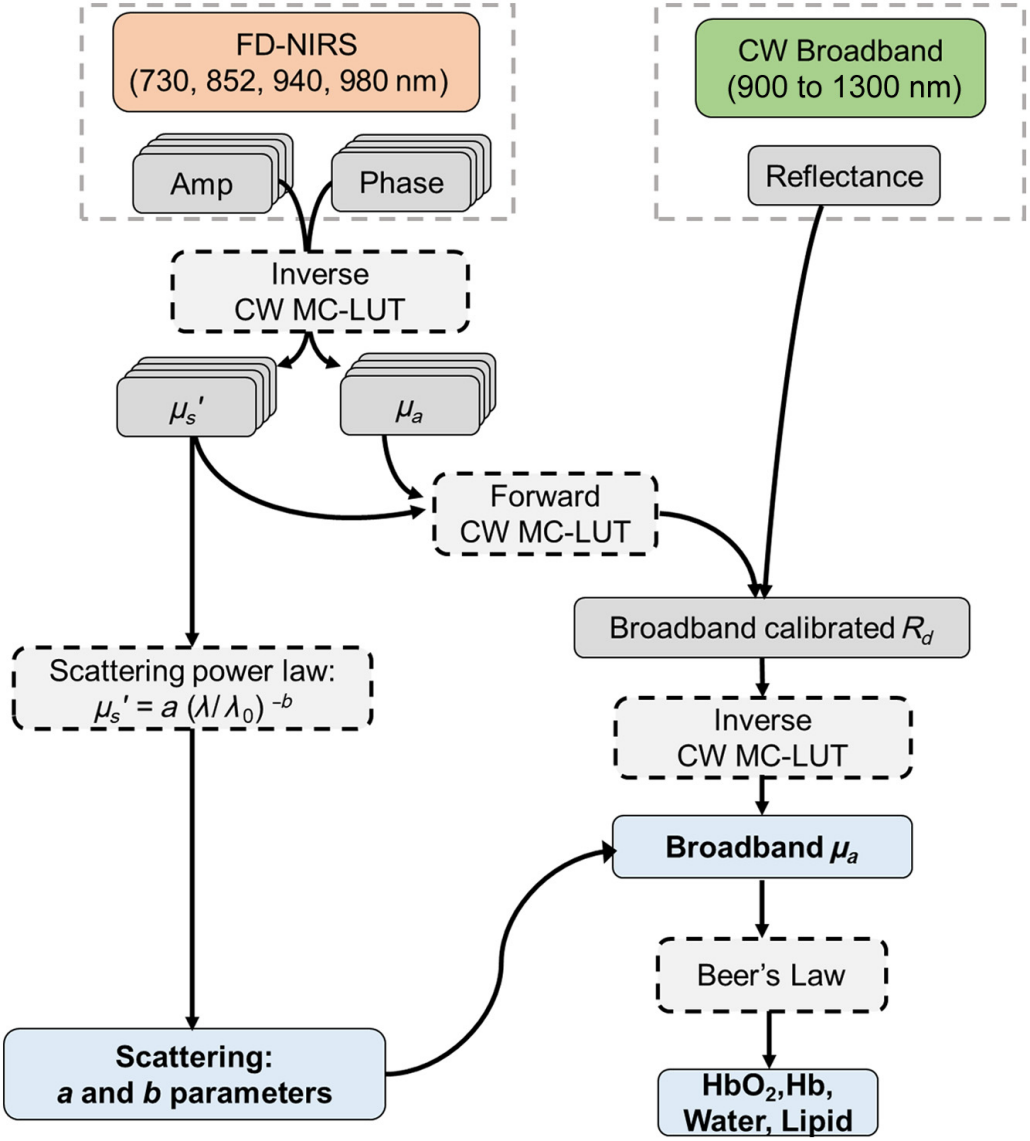
Processing pipeline of the FD-Bb-SWIRS system that combines FD-NIRS and broadband CW-SWIRS systems. Note that $\lambda_{0}=900\;\mathrm{nm}$ for the scattering power law fit.

The processing pipeline for the FD-Bb-SWIRS system is shown in Fig. 4. It is adapted from a previously described algorithm used for combined broadband CW-NIRS and FD-NIRS.^7^ In this case, we leveraged the fact that there is a partial overlap between the FD-NIRS and broadband CW-SWIRS wavelengths to extract $\mu_{a}$ from 900 to 1300 nm. For data processing, we used the MC-LUT approach for a homogeneous medium with semi-infinite geometry to translate optical properties $\mu_{a}$ and $\mu_{s}^{\prime }$ to theoretical reflectance data (forward model) and vice versa (inverse model). The construction of MC-LUT was described in detail in our previous work.^37^^,^^38^ Look-up tables at different modulation frequencies (for FD data) and at 0 MHz (CW MC-LUT for CW data) were generated for the ranges of $\mu_{a}$ from 0 to $0.3\;{\mathrm{mm}}^{-1}$ and $\mu_{s}^{\prime }$ from 0 to $2\;{\mathrm{mm}}^{-1}$. For measurements on intralipid-based liquid phantoms (Sec. 3.2), the refraction index and scattering anisotropy were assumed to be n=1.33 and g=0.7, respectively.^33^ For measurements on solid phantoms and porcine tissues, the values were set as n=1.4 and g=0.9.^24^

First, $\mu_{a}$ and $\mu_{s}^{\prime }$ values at 685 to 980 nm were extracted from the calibrated amplitude and phase values of FD-NIRS measurements using the MC-LUT inverse model. Calibration was conducted using intralipid-based liquid phantoms or solid silicone phantoms. The optical properties of the calibration of intralipid phantoms are known based on our previous work.^33^ Using extracted $\mu_{s}^{\prime }$ at 685 to 980 nm, the wavelength dependence of scattering was fit to a power law,^24^ and the scattering parameters a (scattering amplitude at 900 nm) and b (scattering power) were computed. These scattering parameters were used to obtain spectral $\mu_{s}^{\prime }$ up to 1300 nm.

**Fig. 5:**
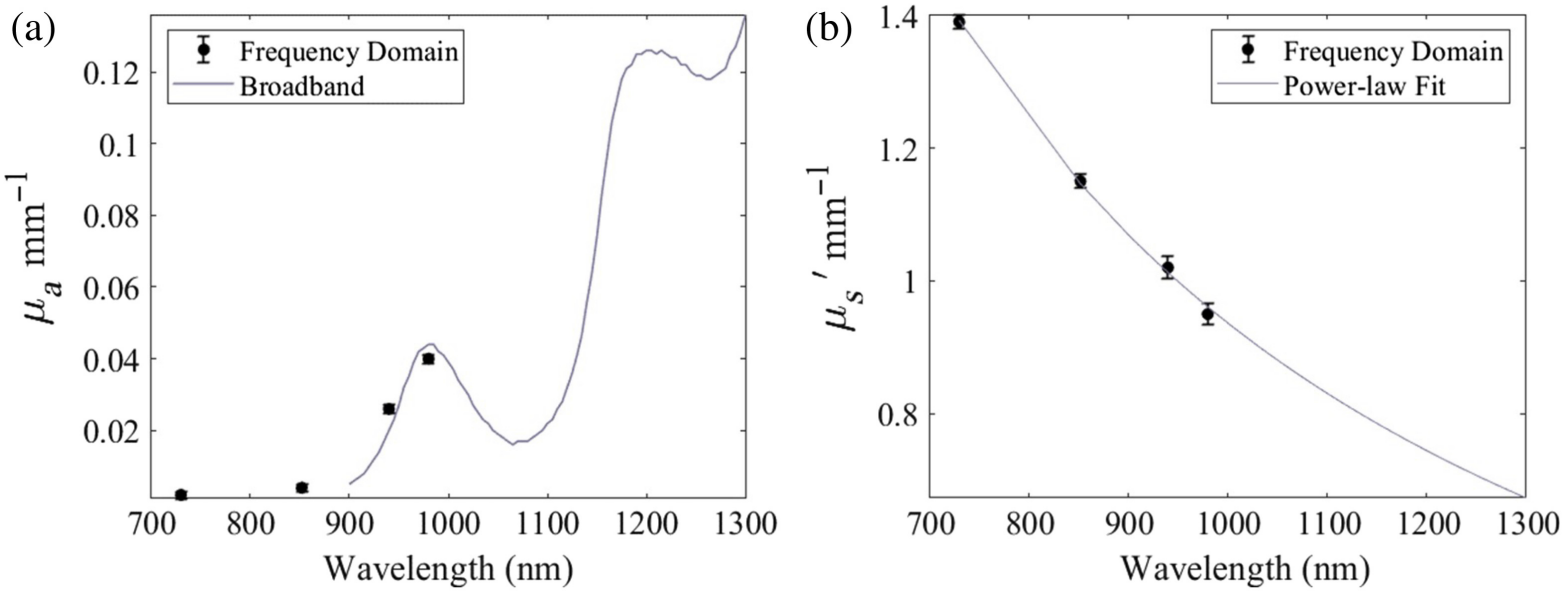
Example FD-Bb-SWIRS measurements of a liquid intralipid phantom composed of 1.5% lipid by volume calibrated against a 1% lipid by volume calibration liquid intralipid phantom. The FDNIRS $\mu_{a}$ (a) and $\mu_{s}^{\prime }$ (b) are overlaid with the respective CW-SWIRS spectra.

Next, broadband $\mu_{a}$ spectra at wavelengths >900  nm were obtained from calibrated CW-SWIRS measurements and known $\mu_{s}^{\prime }$ spectra obtained as in the previous step. The broadband CW-SWIRS measurements were first calibrated using a Spectralon standard (Labsphere, North Sutton, New Hampshire, United States) to correct for spectral features of the lamp light source. The broadband CW-SWIRS reflectance was then scaled to match with the expected CW reflectance values at the overlapping FD wavelengths, as computed from the optical properties at those wavelengths obtained in the first step using the CW MC-LUT forward model. With the calibrated CW reflectance data and known $\mu_{s}^{\prime }$ values at 900 to 1300 nm, we used the CW MC-LUT inverse model to compute $\mu_{a}$ spectrum. Figure 5 shows an example measurement of a liquid phantom composed of intralipid (1.5% lipid by volume), which clearly shows the expected absorption peak of water near 980 and 1200 nm.

For tissue measurements, tissue chromophore concentrations, i.e., $\left[{\mathrm{HbO}}_{2}\right]$, [Hb], [water], and [lipid], were obtained from the broadband $\mu_{a}$ spectra (at FD wavelengths from 685 to 980 nm and CW-wavelengths from 900 to 1300 nm) by applying the least-square fitting method to minimize $\mu_{a}$ spectra and Beer’s law model (Sec. 2.4.3).

### 2.4 Experiments

#### 2.4.1 *In vitro* validations on mineral oil–based phantoms

In the first experiment, the FD-Bb-SWIRS system was used to measure solid mineral oil–based phantoms with different absorption values to determine the system’s ability to measure $\mu_{a}$ spectra up to 1300 nm. Solid mineral oil–based phantoms were fabricated following the method described in Hacker et al.^39^ In this protocol, we substituted nigrosin dye with powdered carbon black dye (Jacquard Products, Healdsburg, California, United States). Carbon black has flat $\mu_{a}$ spectra across the NIR and SWIR wavelength ranges,^40^ whereas the nigrosin $\mu_{a}$ spectrum decreases with wavelength.^41^ Three 125-mL phantoms were fabricated with increasing carbon black dye concentrations: 0.05, 0.08, and 0.16  g/L. Each phantom contained 100 mL of mineral oil (Sigma Aldrich, St. Louis, Missouri, United States), 0.15 g of titanium dioxide (Sigma Aldrich, St. Louis, Missouri, United States), 25.14 g of SEBS [polystyrene-block-poly(ethylene-ran-butylene)] (Sigma Aldrich, St. Louis, Missouri, United States), and 6.70 g of low-density polyethylene (Alfa Aesar, Haverhill, Massachusetts, United States). Measurements were taken with an SDS of 10 mm. The FD measurements taken in this experiment utilized wavelengths at 685-, 730-, 852-, 915-, and 980-nm FD wavelengths. However, data at 730 and 915 nm were excluded due to phase noise >2  deg or amplitude noise >2%. The FD measurements were calibrated against a solid silicone calibration phantom (namely, ACRIN9) with known optical properties.

#### 2.4.2 *In vitro* validations on $D_{2}O$ liquid phantoms

In the second experiment, the FD-Bb-SWIRS system was used to measure liquid intralipid–based phantoms fabricated from a consistent concentration of intralipid solution (Patterson Veterinary, Houston, Texas, United States) and at different concentrations of deuterium oxide ($D_{2}O$) (Sigma Aldrich, St. Louis, Missouri, United States) to test the feasibility of the system to measure changes in $\mu_{a}$ around the water absorption peaks at 980- and 1200-nm wavelengths. Measurements were taken from a solution of 1% lipids by volume held within a 10  cm×7  cm×2.5  cm opaque well, able to hold 150 mL of fluid. The lipid solution was incrementally diluted with a similar mixture of 1% lipids and $D_{2}O$ over 10 titration steps of increasing $D_{2}O$ from 0% to 90% $D_{2}O$. The solution was measured five times per titration step. Measurements were taken with an SDS of 10 mm at 730-, 852-, 940-, and 980-nm FD wavelengths. FD measurements were calibrated against a separate 1% by volume lipid and deionized (DI) water solution. $D_{2}O$ has ∼1/10th the $\mu_{a}$ of DI water in the SWIR wavelength range.^42^ Thus, as the $D_{2}O$ replaced $H_{2}O$ in the solution, it was anticipated that the $\mu_{a}$ would decrease while $\mu_{s}^{\prime }$ remained constant.^10^

#### 2.4.3 *Ex vitro* validations on porcine samples during desiccation

**Fig. 6:**
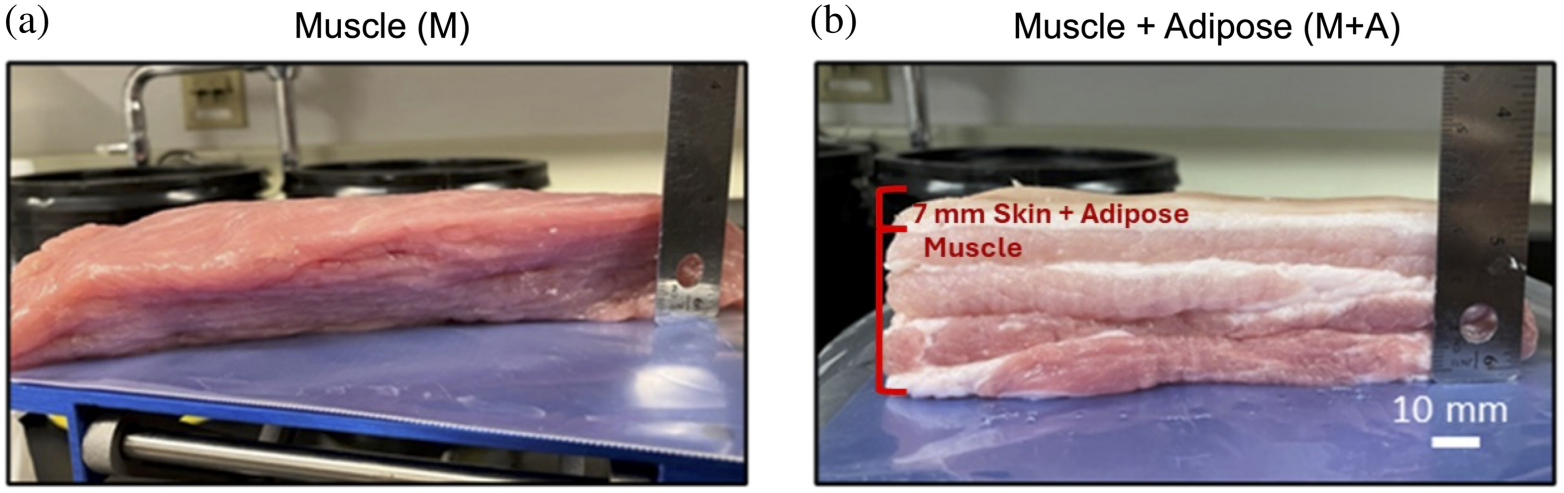
(a) and (b) Photos of the two porcine samples, muscle (M) sample and muscle with an adipose layer (M+A) sample. Photos were taken at baseline (before desiccation).

In the final experiment, the FD-Bb-SWIRS system was validated in *ex vivo* measurements on porcine tissue to assess the ability of the system to monitor water changes in tissue. Measurements were performed on two different porcine tissue samples, a lean muscle (M) sample and a sample with a 7-mm adipose + skin layer on top of the muscle (muscle + adipose or M+A) (Fig. 6). The samples were acquired fresh and refrigerated. Before measurements, they were left at room temperature for one hour, assuming they had reached room temperature before the start of the experiment. In this experiment, the SDS was set at 15 mm to allow for deeper light penetration. FD measurements were taken at 730-, 852-, and 915-nm wavelengths. Data at 685 and 980 nm were excluded as phase noise was >2  deg or amplitude noise was >2%. Calibration of FD measurements was performed on a solid silicone calibration phantom (labeled as INO9) with known optical properties. The porcine samples were measured with the FD-Bb-SWIRS system in three locations by moving and replacing the probe between each measurement. These locations were marked for subsequent measurements. For the M+A sample, measurements were taken on top of the skin layer. After baseline measurements, the porcine samples were placed inside an electric vacuum oven at 30°C to 40°C for the next 8-h desiccation period. The specified temperature range was selected to allow for continuous evaporation of water while not inducing protein denaturation.^43^ Over the course of 8 h, the porcine samples were removed once an hour to be weighed and optically measured with the FD-Bb-SWIRS system in the same locations as the baseline measurements. After 10 h, the porcine sample continued to undergo desiccation and was weighed every 12 h until no further weight loss was observed due to water evaporation (after 177 h in total). The water content at each desiccation time point x was calculated based on weight loss relative to the final recorded weight,^44^ as shown in Eq. (1):

$$C_{x}=\frac{m_{x}-m_{c}}{m_{x}}\times 100\;(\%).$$ (1)

Here, $C_{x}$ is the water content of the porcine sample at time x, $m_{x}$ is the measured mass after x hours of desiccation, and $m_{c}$ is the final mass of the porcine sample recorded at the end of the desiccation period (i.e., after 177 h).

The $\mu_{a}$ spectra measured at the three locations for each desiccation time point were averaged to get tissue chromophore concentrations ($\left[{\mathrm{HbO}}_{2}\right]$, [Hb], [water], and [lipid]). The fitting method for chromophore concentrations was carried out including a constant background absorption. Specifically, the model indicates that $\mu_{a}$ spectrum is dependent on the concentrations (C) and respective extinction coefficient spectra (ε) of the chromophores, such that $\mu_{a}(\lambda )=\underset{i}{\sum }C_{i}\varepsilon_{i}(\lambda )+\mu_{a,bkg}$. Here, a constant background absorption $\mu_{a,bkg}$ was added to account for tissue heterogeneity or missing chromophores.^22^ Extinction coefficients ε of the four chromophores are based on values from the literature, listed in the Appendix. In this experiment, we compared the water content extracted from our FD-Bb-SWIRS method with estimates derived from weight loss [using Eq. (1)] and those obtained from FD-Bb-NIRS data (i.e., fitting was performed on the same optical data up to 1000 nm). This comparison aimed to highlight the advantages of the proposed FD-Bb-SWIRS over NIRS in terms of water-fitting accuracy.

## 3 Results

### 3.1 Uncertainty in Extracted Optical Properties Using FD Model

**Fig. 7:**
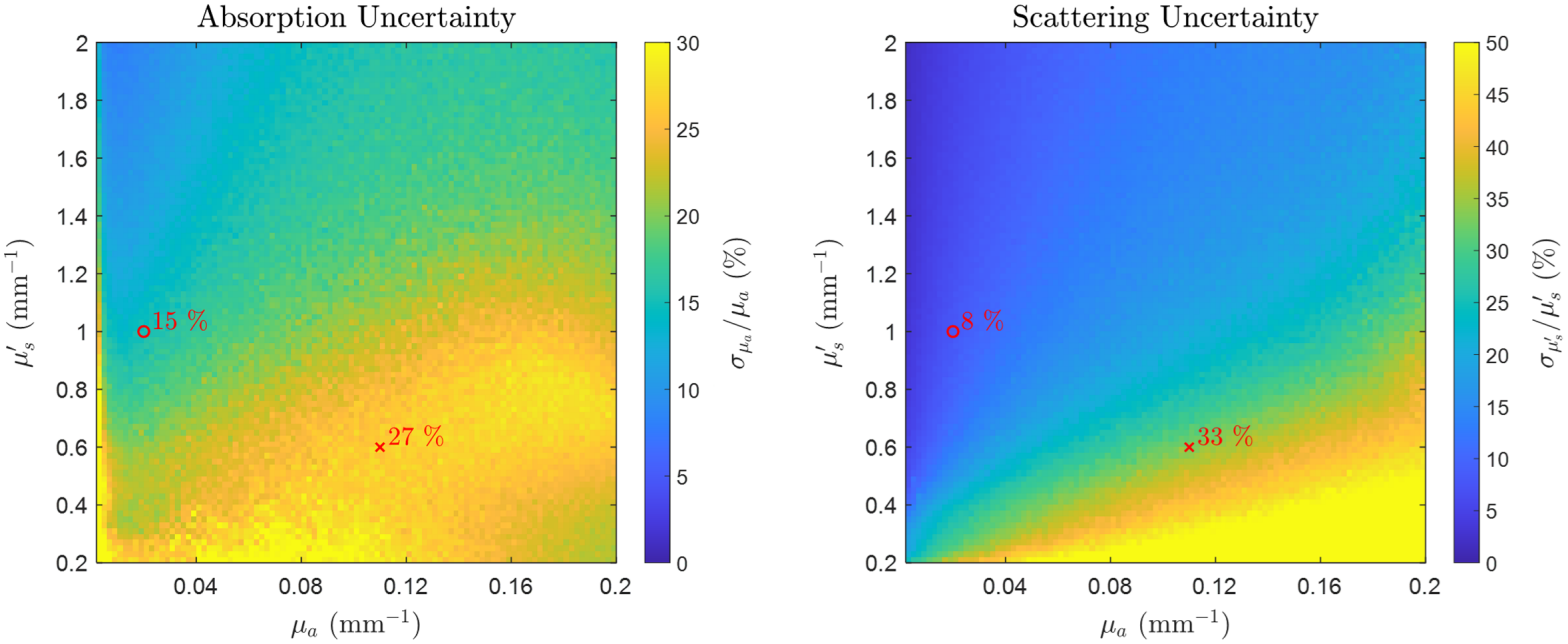
Uncertainties in absorption ($\mu_{a}$) and reduced scattering ($\mu_{s}^{\prime }$) properties obtained from simulations with FD reflectance data. Optical property examples are shown for human muscle tissue at 800 nm ($\mu_{a}=0.02\;{\mathrm{mm}}^{-1}$, $\mu_{s}^{\prime }=1\;{\mathrm{mm}}^{-1}$, shown as a red “o” symbol) and 1200 nm ($\mu_{a}=0.11\;{\mathrm{mm}}^{-1}$, $\mu_{s}^{\prime }=0.6\;{\mathrm{mm}}^{-1}$, shown as a red “x” symbol). These plots are generated from values obtained from typical muscle tissue chromophore concentrations (total hemoglobin ([HbT])=117  μM, oxygen saturation $({\mathrm{StO}}_{2})=65\%$, [water]=60%, and [lipid] = 20%) and scattering parameters ($\mu_{s}^{\prime }\;(500\;\mathrm{nm})=1.5\;{\mathrm{mm}}^{-1}$, b=0.9).^23^^–^^32^

Figure 7 illustrates the simulated uncertainties of the extracted $\mu_{a}$ and $\mu_{s}^{\prime }$ when using FD reflectance data obtained at different optical property values. When subjected to the same noise level in FD reflectance data, we observed increased variances in the recovered $\mu_{a}$ and $\mu_{s}^{\prime }$ with increasing $\mu_{a}$. Absorption uncertainty was notably higher in the region with high $\mu_{a}$ and low $\mu_{s}^{\prime }$ (SWIR regime). In particular, we presented two sets of optical properties representing typical values for human muscle tissue at 800 nm (NIR) and 1200 nm (SWIR). Results indicated that the absorption uncertainty $\sigma_{\mu_{a}}/\mu_{a}$ was approximately twice as high at 1200 nm compared with 800 nm. Similarly, scattering uncertainty $\sigma_{\mu_{s}^{\prime }}/\mu_{s}^{\prime }$ was about four times greater at 1200 nm than at 800 nm. The increased uncertainties in optical properties at 1200 nm are further shown by the higher standard deviations observed in the distributions of the extracted $\mu_{a}$ and $\mu_{s}^{\prime }$, as shown in Figs. S1(A) and S1(B) in the Supplementary Material. In addition, the differences between the means of these extracted optical property distributions and the simulated ground truth values are notably higher for high $\mu_{a}$ and low $\mu_{s}^{\prime }$ values but remain relatively uniform otherwise [see Figs. S1(C) and S1(D) in the Supplementary Material].

The simulation results underscore the challenge of obtaining accurate optical property measurements using only FD technology in the SWIR, in contrast to the NIR. This serves as motivation for this study to employ a combination of FD-NIRS and broadband CW-SWIRS techniques to improve the accuracy of optical property measurements.

### 3.2 *In Vitro* Validations on Mineral Oil–Based Phantoms

**Fig. 8:**
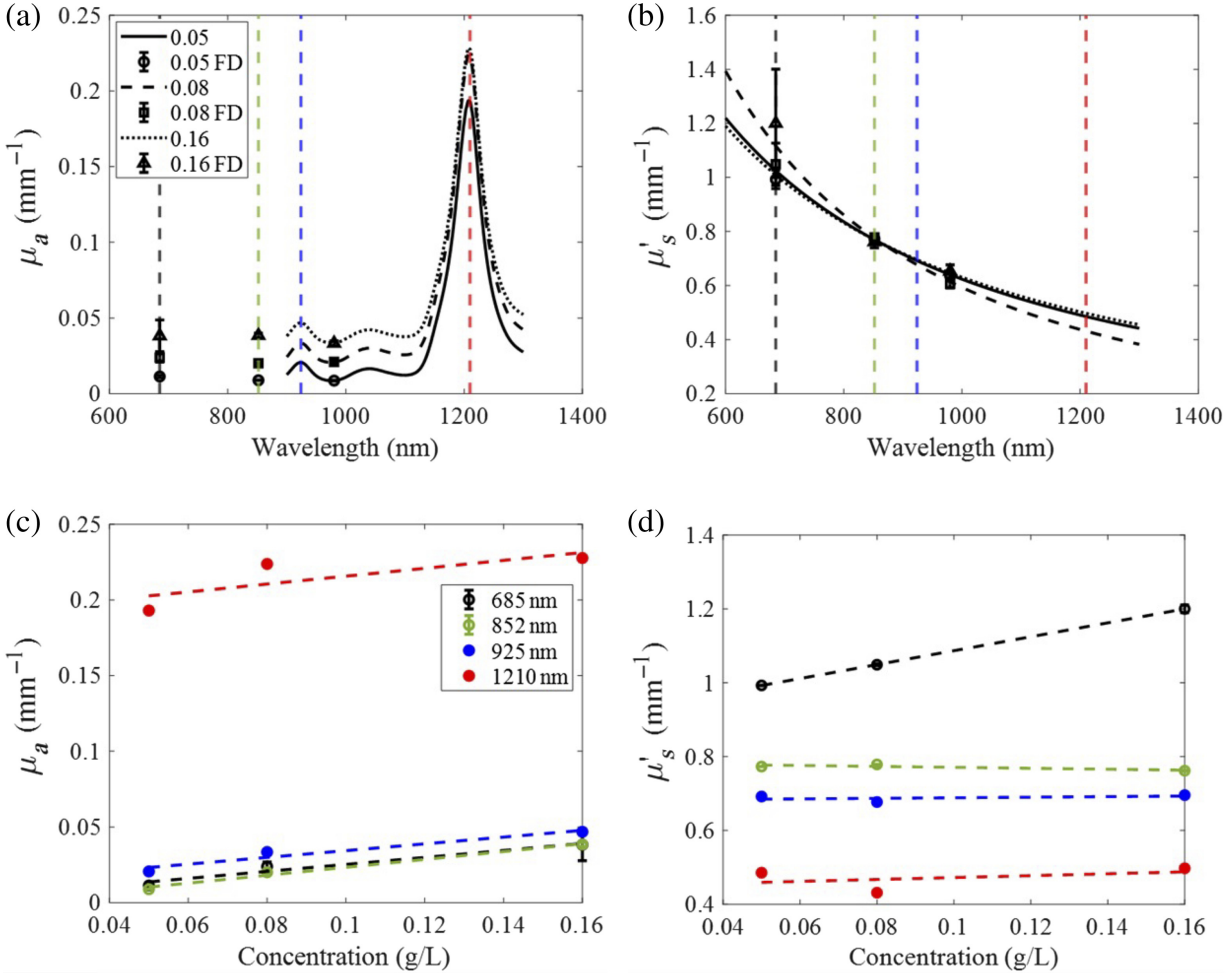
Spectra of (a) $\mu_{a}$ and (b) $\mu_{s}^{\prime }$ of three solid mineral oil–based phantoms with carbon black concentrations at 0.05, 0.08, and 0.16  g/L. Values of $\mu_{a}$ (c) and $\mu_{s}^{\prime }$ (d) at 685, 852 (from FD measurements), 925, and 1210 nm (from broadband extrapolated values) versus the different carbon black concentrations in the phantoms. Lines in panels (c) and (d) indicate the linear fits across all data points.

Figures 8(a) and 8(b) show $\mu_{a}$ and $\mu_{s}^{\prime }$ spectra obtained from the three solid mineral oil–based phantoms. FD measurements of $\mu_{a}$ and $\mu_{s}^{\prime }$ are shown as means and standard deviations averaged from 300 sequential measurements without probe repositioning. As anticipated, $\mu_{a}$ increased linearly with increased carbon black concentration [Figs. 8(a) and 8(c)], whereas $\mu_{s}^{\prime }$ remained nearly constant [Fig. 8(b)]. Though there was some fluctuation in $\mu_{s}^{\prime }$ over the increasing carbon black concentration, there is no singular trend as seen in Fig. 8(d), and thus, any drift in the values can be attributed to instrumental/experimental noise or slight variations in phantom creation. All presented measurements were below the 2-deg phase error and 2% amplitude error. Lipid absorption peaks at 925 and 1210 nm were present in all phantoms, which is attributable to the previously reported spectra of mineral oil.^45^ The broadband $\mu_{a}$ featured a prominent SWIR lipids peak at 1210 nm, approximately five times the magnitude of the peak at 925 nm. The slope values of $\mu_{a}$ changes versus carbon black concentrations shown in Fig. 8(c) are 0.23, 0.26, 0.22, and $0.26\;{\mathrm{mm}}^{-1}/(g/L)$ for 685, 852, 925, and 1210 nm, respectively.

### 3.3 *In Vitro* Validations on $D_{2}O$ Liquid Phantoms

**Fig. 9:**
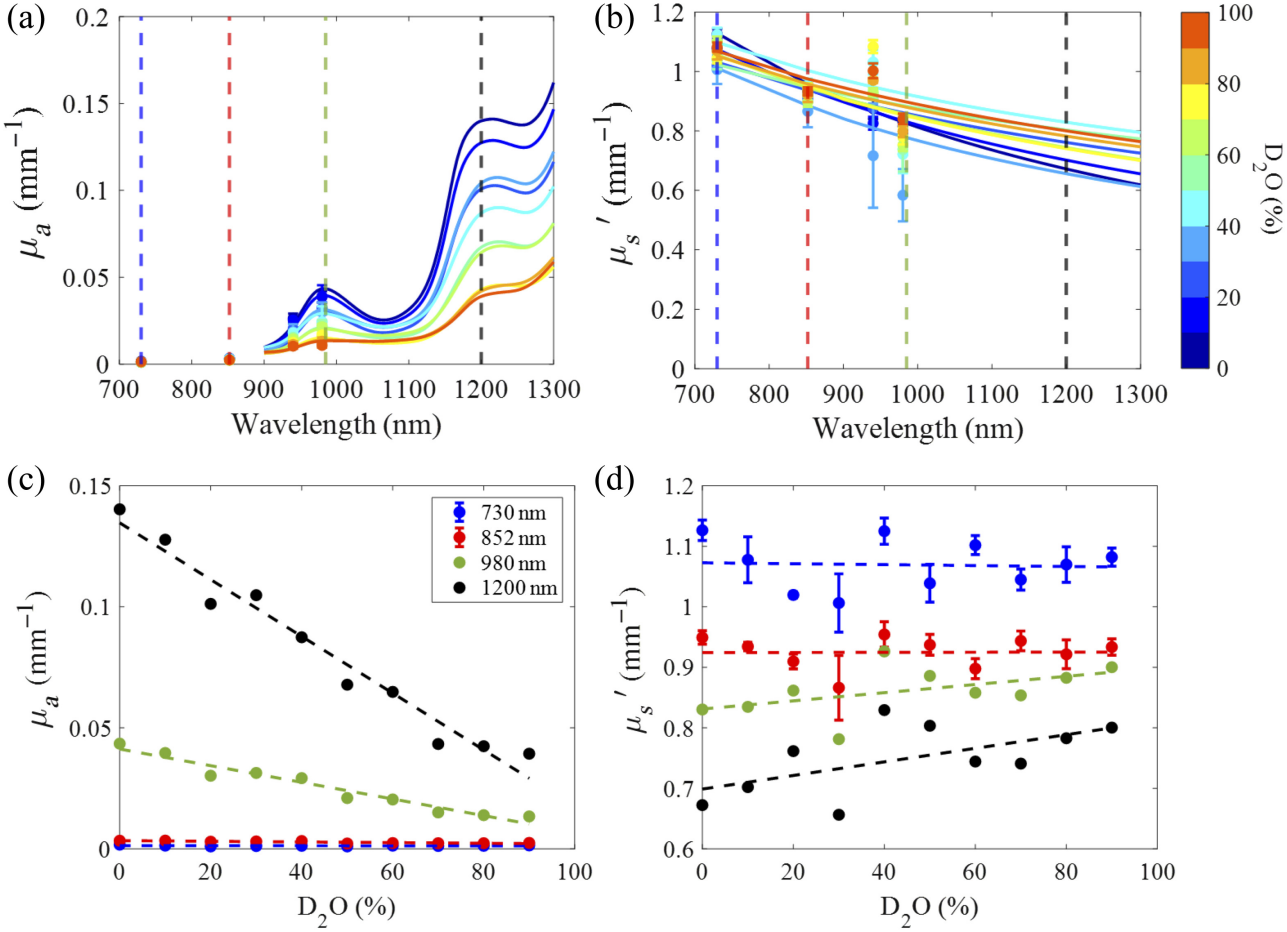
Spectra of (a) $\mu_{a}$ and (b) $\mu_{s}^{\prime }$ of the 10 titration steps. The titration was performed from 0% $D_{2}O$ to 90% $D_{2}O$. Values of $\mu_{a}$ (c) and (d) $\mu_{s}^{\prime }$ at 730, 852 (from FD measurements), 980, and 1200 nm (from broadband measurements) versus the different $D_{2}O$ concentrations. Lines in panels (c) and (d) indicate the linear fits over all data points.

Figure 9 shows the broadband $\mu_{a}$ and $\mu_{s}^{\prime }$ values extracted from the $D_{2}O$ titration. As anticipated, $\mu_{a}$ values decreased linearly around the water absorption peaks of 980 and 1200 nm [Fig. 9(a)], whereas $\mu_{s}^{\prime }$ values remained unchanged as $D_{2}O$ concentrations increased [Figs. 9(b) and 9(d)].

Figure 9C shows $\mu_{a}$ values at 730, 852, 980, and 1200 nm measured at different $D_{2}O$ concentrations. This figure shows that the slopes of $\mu_{a}$ changes at 730 and 852 nm over $D_{2}O$ titration steps are very small (close to zero). The slopes of $\mu_{a}$ changes at 980 and 1200 nm (associated with water absorption peaks) were significantly larger, i.e., −0.03 and $-0.12\;{\mathrm{mm}}^{-1}/100\%$ $D_{2}O$ for 980 and 1200 nm, respectively. Notably, the maximum $\mu_{a}$ peak at 1200 nm is approximately three times greater than the peak at 980 nm. Though there was some fluctuation in $\mu_{s}^{\prime }$ values over the 10 titration steps, there is no singular trend [see Fig. 9(d)], and thus, any drift in the values may be attributed to instrument or experimental noise.

### 3.4 *Ex Vitro* Validations on Porcine Samples During Desiccation

**Fig. 10:**
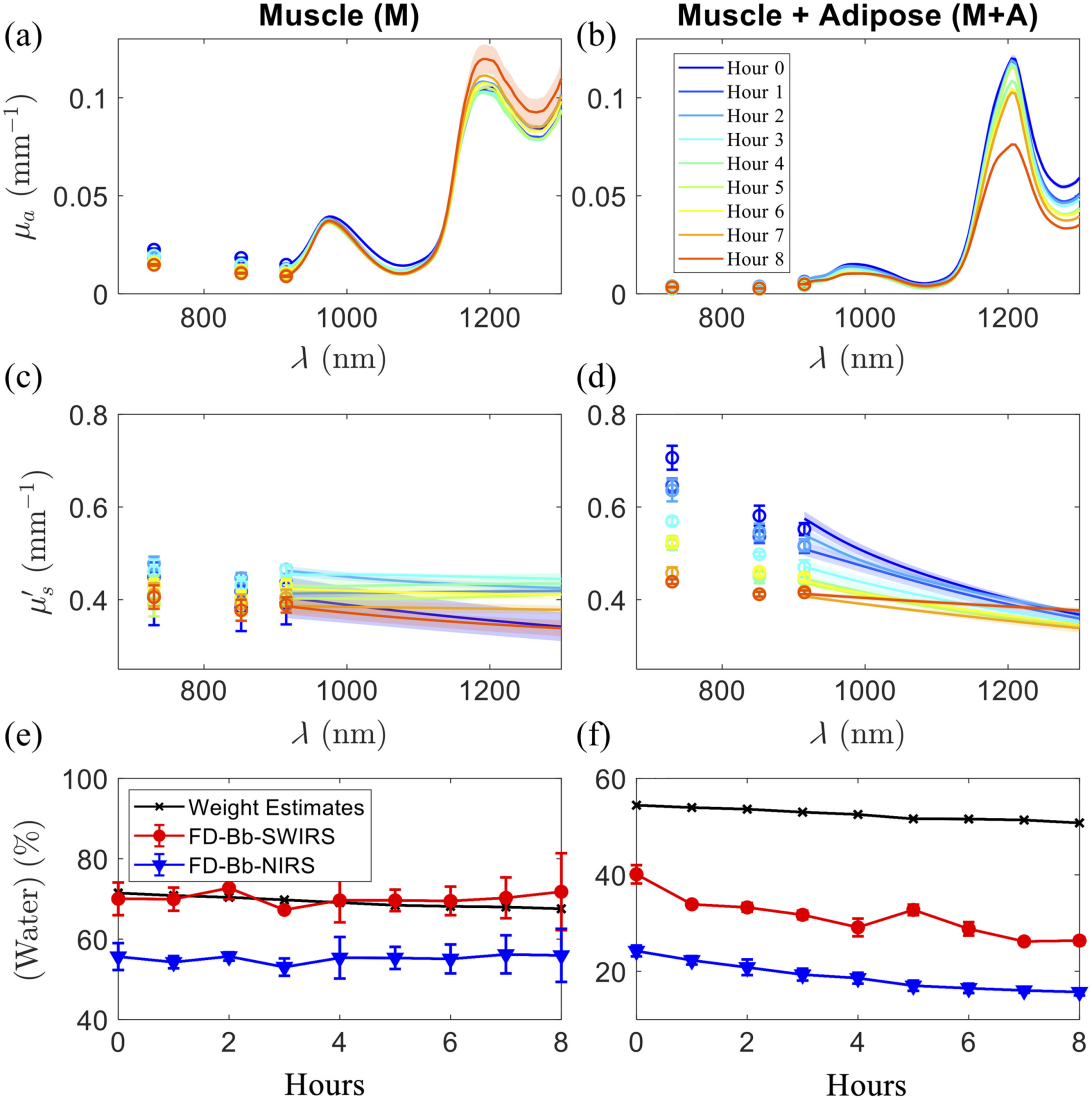
Optical properties and water extraction results for two porcine tissues: a muscle (M) sample and a muscle with adipose layer (M+A) sample. Data were collected every hour over an 8-h desiccation period. Absorption ($\mu_{a}$) spectra (a) and (b) and reduced scattering ($\mu_{s}^{\prime }$) spectra (c) and (d) are presented for each desiccation hour, with values shown as means and standard errors based on three separate location measurements. Panels (e) and (f) show the extracted water concentrations (water) over time for both samples, derived from weight estimates, FD-Bb-SWIRS optical data, and FD-Bb-NIRS optical data (up to 1000 nm). The results are shown with means and standard errors from measurements taken at three distinct locations.

Figure 10 presents $\mu_{a}$ spectra measured from two porcine samples, one consisting solely of muscle (M) and the other with a layer of 7-mm adipose + skin at the top of the muscle (M+A). Figure 10 also presents the tissue water concentrations [water] derived from weight estimates [using Eq. (1)] FD-Bb-SWIRS optical data and FD-Bb-NIRS optical data (up to 1000 nm) for the two porcine samples during the desiccation. Measurements were taken from three distinct locations, with the probe being moved and repositioned between each reading. The $\mu_{a}$ spectra were fit to Beer’s law to extract chromophore concentrations including a constant background absorption. The raw intensity measurements for the porcine samples are discussed in the Supplementary Material, Sec. 2.

At baseline (prior to desiccation), the proposed FD-Bb-SWIRS method measured [water] as 70±4% for the M sample and 40±2% for the M+A sample. The extracted [lipid] was 9.3±0.5% for the M sample and 45.8±0.7% for the M+A sample. The constant absorption background $\mu_{a,bkg}$ was $0.003\pm 0.003\;{\mathrm{mm}}^{-1}$ for the M sample and $1.7\pm 0.1\times 10^{-6}\;{\mathrm{mm}}^{-1}$ for the M+A sample. From baseline to the end of desiccation, [lipid] measured by FD-Bb-SWIRS increased slightly in the M sample (from 9.3% to 11.9% on average) and remained relatively stable in the M+A sample (from 45.8% to 44.7% on average). Over the 8-h desiccation period, the M sample exhibited minimal variation in the measured [water], increasing slightly from an average of 70% at baseline to 72% after 8 h. This negligible change closely aligned with weight loss–based estimates [using Eq. (1)], which indicated 71% at baseline and 68% after 8 h. In contrast, the M+A sample showed a more pronounced decrease in FD-Bb-SWIRS measured [water], dropping from an average of 40% at baseline to 26% after 8 h. These measured values differed from the estimated water content from weight loss, which indicated 54% at baseline and 51% after 8 h. Throughout the desiccation process, the FD-Bb-NIRS method consistently underestimated water content compared with both the FD-Bb-SWIRS chromophore extractions and the weight loss–based estimates in both tissue samples.

The discrepancy in estimated water content between FD-Bb-SWIRS and weight estimates for the M+A sample is likely due to the relatively small 15-mm source-detector distance, which likely resulted in increased sensitivity to the skin and adipose layers while providing much less sensitivity to the muscle layer.^46^^,^^47^ Thus, the lower [water] values measured in the M+A sample may be attributed to the lower water content in adipose tissue compared with muscle.^48^^–^^50^ In contrast, the weight loss–based water content estimates for the M+A sample likely reflect contributions from both muscle and adipose tissues.

## 4 Discussion

We presented here a combined FD and CW system to measure $\mu_{a}$ and $\mu_{s}^{\prime }$ from 685 to 1300 nm. To the best of our knowledge, this is the first combined FD and CW system capable of quantifying water and lipids in the SWIR regime (i.e., >1000  nm).

The system was shown to be sensitive to $\mu_{a}$ changes in mineral oil–based phantoms with increasing concentrations of carbon black. A similar trend in $\mu_{a}$ and carbon black concentration was observed in Maneas et al.^51^ in which $\mu_{a}$ increased linearly with carbon black concentration. The mineral oil–based phantoms also exhibited strong $\mu_{a}$ peaks at 925 and 1210 nm, characteristic of lipid absorption. In addition, the FD-Bb-SWIRS system was shown to be sensitive to absorption changes due to water content in the $D_{2}O$ titration experiment. Here, prominent absorption peaks were observed at 980 and 1200 nm, characteristic of water absorption.

The system was also able to quantify changes in $\mu_{a}$ and $\mu_{s}^{\prime }$, as well as water and lipid concentrations in *ex vivo* porcine tissues. To the best of our knowledge, this paper is the first to compare NIR versus SWIR optical windows in a hybrid FD and CW broadband system in terms of the accuracy of water content extraction in biological tissue. Our results demonstrated that the proposed FD-Bb-SWIRS system provided better estimations of tissue water content measured by weight loss–based estimates. In this *ex vivo* experiment, both lean muscle (M) and fatty muscle + adipose (M+A) porcine tissues were measured, which had substantially different properties and trends during desiccation. Optical measurements showed lower water content at baseline values in M+A than in M sample. This could be explained because in the M+A tissue, measurements were sensitive to the more superficial skin and adipose layers where there is a lower water content than in muscle.^50^ Our optical measurements on M and M+A samples showed similar spectral features to those measured on porcine muscle and pure fat tissue samples, respectively, as reported in Damagatla et al.^23^ Specifically, the M sample showed clear water absorption peak at 980 and a broad peak at 1200 nm, whereas the M+A sample showed a more prominent and narrow lipid absorption peak at 1210 nm. Mitchell et al.^52^ reported a muscle water content of 73±0.5% which is similar to our measurements of 70%±4% for the M sample at baseline. Lam et al.^9^ reported averaged water contents of 43% and 56% for porcine tissue samples with thick and thin adipose tissue layers, respectively. Our water content estimates for our M+A measurements are at 40%±2% aligning within the range of values reported from Lam et al. After 8 h of desiccation, water changed more significantly in the M+A sample (about an average of 14%) as compared with the M sample. This may be because the superficial tissues (e.g., skin and adipose) probed by our optical measurements may undergo different proportional changes in water content during the desiccation process compared with pure muscle tissue, when compared with prior dehydration assessments of porcine skin tissue our findings report similar trends. In Yu et al.,^44^ there is a marked decrease of ∼15% in water over the first 8 h of dehydration of a porcine dermis sample, which we see similar trends to in the M+A sample.

Compared with prior SWIR measurement techniques, the FD-Bb-SWIRS system presented here has several important and distinct characteristics. Importantly, the use of FD measurements provided absolute quantification of tissue optical properties both at the FD measurement wavelengths and then from 900 to 1300 nm through scaling of our broadband CW spectroscopy data. In contrast, our prior work in the development of a CW wearable SWIR probe utilized multiple SDS as well as a deep neural network to extract water and lipid concentrations, without direct extraction of optical properties.^10^ Similarly, other works such as Nachabé et al.^8^ utilized spectral shape constraints to fit for scattering parameters and tissue chromophores directly without extracting tissue absorption. Our method allows for more direct extractions of chromophores, such as water and lipids, from absorption measurements without the need for spectral constraint assumptions on raw optical data. Nachabé et al. also differed from our own in that it used only CW measurements and utilized a very short SDS of 2.48 mm, whereas our system used either 10 or 15 mm SDS allowing for measurements of deeper tissue regions. In addition, our measurements utilized both NIR and SWIR wavelength ranges so that concentrations of blood oxy- and deoxyhemoglobin could be included in our chromophore fits in future work.

In comparison with prior works utilizing the NIR wavelength band to extract tissue water and lipids concentrations,^9^^,^^19^^,^^53^ our method captures both the first and second lipid and water absorption peaks. As expected from the known extinction values, our findings confirmed that the 1200-nm absorption peak is more sensitive to changes in water and lipids compared with the 900- to 1000-nm range. For example, in the $D_{2}O-H_{2}O$ titration, the slope of the relationship between $H_{2}O$ concentration and $\mu_{a}$ was approximately four times steeper at 1200 nm compared with 980 nm [Fig. 9(c)]. This is likely to assist in the detection of more subtle changes in tissue water and lipid concentrations.

We have previously reported on extending SFDI to the SWIR wavelength band.^11^^,^^33^^–^^35^ SFDI is non-contact and uses spatial modulation to extract quantitative optical properties over a wide field. One major difference between SWIR-SFDI measurements and the FD-Bb-SWIRS measurements presented here is the depth of penetration. SFDI has a relatively shallow penetration depth, typically ranging from a few hundred microns to ∼3  mm.^11^^,^^33^ In contrast, the direct-contact configuration and spatially separated source and detector fibers used in FD-Bb-SWIRS potentially allow for deeper tissue measurements, reaching up to ∼8  mm inside the tissue for the SDS of 15 mm used in the *ex vivo* experiments.^9^^,^^37^

The ability to non-invasively quantify water and lipid content using FD-Bb-SWIRS may be important for several clinical scenarios. For example, the volume status of patients receiving hemodialysis is not currently directly assessed, and it is estimated that 20% to 50% of all dialysis patients are inadequately dialyzed.^14^^,^^15^^,^^54^^,^^55^ Direct measurements of tissue water content with FD-Bb-SWIRS might help with the management of the administration of dialysis for these patients. Similarly, this or similar technology could assist with monitoring fluid overload and edema in patients with cardiovascular disease, especially heart failure.^16^^,^^56^ This technique may also be useful for non-invasive quantification of hydration level and body composition,^49^ such as monitoring intentional weight gain or loss, or in sports medicine.^18^ Finally, changes in lipid concentrations have been shown to relate to chemotherapeutic response in breast cancer.^12^^,^^13^^,^^21^^,^^53^^,^^57^

One limitation of this work was the use of a relatively small SDS of 15 mm or less. This was primarily due to the inability to collect useable measurements near the strong 1200-nm absorption peak of tissue at longer SDS. In the future, this might be improved with higher illumination power near this wavelength band and/or improved detector sensitivity. Another limitation was the use of homogenous MC models, which are appropriate for homogenous liquid phantoms, but fail to capture the layered structure of tissue, including the skin, adipose, and muscle.^33^^,^^58^

## 5 Conclusion

In summary, in this work, we designed and fabricated an FD-Bb-SWIR device for the characterization of tissue optical properties up to 1300 nm. The system’s sensitivity to changes in $\mu_{a}$ at both the water and lipid absorption peaks in the NIR and SWIR was validated through both solid and liquid phantom titrations in the carbon black and $D_{2}O$ experiments. In addition, the system’s ability to extract water concentration changes *ex vivo* was showcased via chromophore extractions during a porcine desiccation experiment. In the future, this technique has the potential to provide sensitive water and lipid measurements for a variety of clinical applications.

## 6 Appendix: Chromophore Extinction Coefficients

The sources of tissue chromophore extinction coefficients used in this paper are as follows:

${\mathrm{HbO}}_{2}$ **4 and Hb (430 to 599 nm)**: Scott Prahl OMLC,^59^ scaled to match with those at 600 nm.
${\mathrm{HbO}}_{2}$ **5 and Hb (600 to 1000 nm)**: Zijlstra et al. (*Visible and Near Infrared Absorption Spectra of Human and Animal Haemoglobin determination and application*, First Edition, 2000) and Kou et al. (*Applied Optics*, 1993).
${\mathrm{HbO}}_{2}$ **and Hb (1001 to 2000 nm)**: Kuenstner and Norris (*Journal of Near Infrared Spectroscopy*, 1994), scaled to match with those at 1000 nm.
**Water (430 to 559 nm)**: Pope and Fry et al. (*Applied Optics*, 1997).
**Water (600 to 2000 nm)**: Seglestein et al. (1981). Values overlap very well with Pope and Frye and no scaling was needed to match the two datasets.
**Lipids (430 to 1098 nm)**: van Veen and Sterenborg et al. [*OSA Technical Digest* (Optica Publishing Group), 2004].
**Lipids (1100 to 2000 nm)**: Anderson et al. (*Lasers Surg. Med.*, 2006).
**Water and lipids (900 to 1600 nm)**: Nachabé et al. (JBO, 2010).

## Supplementary Material

10.1117/1.JBO.30.4.045001.s01

## Data Availability

All relevant code, data, and materials are available from the authors upon reasonable request. Correspondence and requests should be addressed to the corresponding author.
