# Supplementary material for: Frequency domain broadband short-wave infrared spectroscopy for measurement of tissue optical properties from 685 to 1300 nm
Source: J Biomed Opt. 2025 Apr 25;30(4):045001. doi: 10.1117/1.JBO.30.4.045001 (PMC12022801; doi:10.1117/1.JBO.30.4.045001)
Supplement: Supplementary file 1 [file JBO_030_045001_SD001.docx]

**1. Simulations of extracted optical property uncertainties using FD model**

**Fig S1**. Examples of extracted absorption ($\mu_{a}$, panel **A**) and reduced scattering ($\mu_{s}^{'}$, panel **B**) distributions, showing for two sets of optical properties representing values for human muscle tissue at 800 nm ($\mu_{a}=0.02 \mathrm{mm}^{-1}, \mu_{s}^{'}=1 \mathrm{mm}^{-1}$, shown in blue) and 1200 nm ($\mu_{a}=0.11 \mathrm{mm}^{-1}, \mu_{s}^{'}=0.6 \mathrm{mm}^{-1}$, shown in red). Ground truth values are indicated by solid lines, while the distribution mean values are shown in dashed lines. Panels **C** and **D** display the absolute differences between the ground truth values and the distribution means across different $\mu_{a}$ and $\mu_{s}^{'}$ values.

**2. Ex Vivo Porcine Raw Intensity Measurements**

**
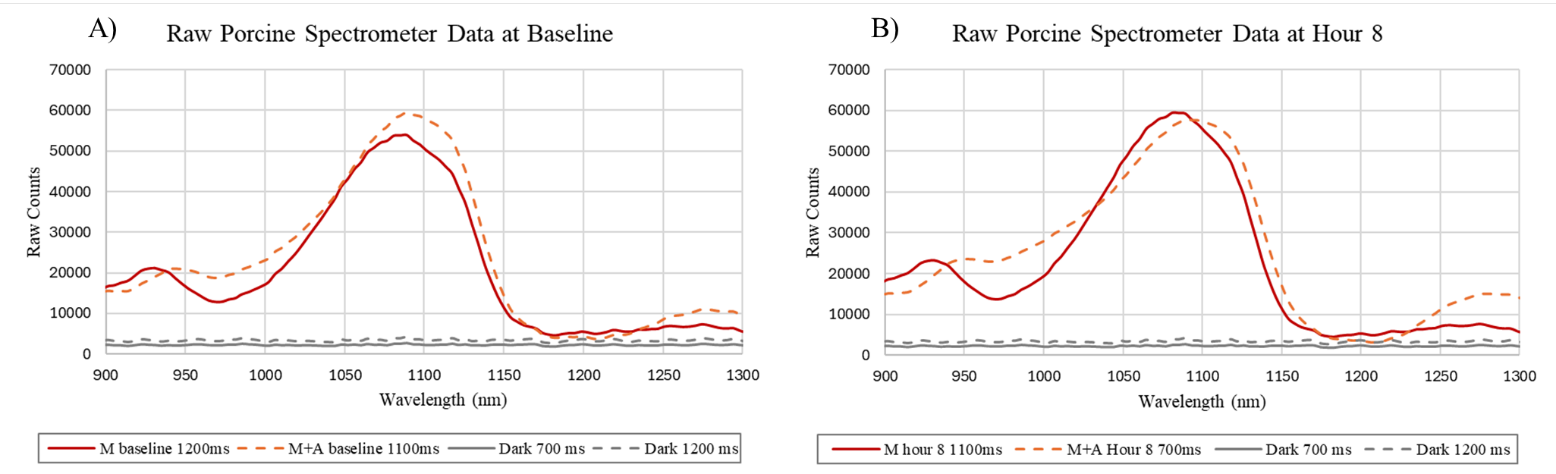
**

**Fig. S2** Example raw spectra at **A)** Baseline and **B)** Hour 8 for both M and M+A porcine samples as well as dark phantoms at similar integration times. All porcine sample spectrometer counts were several hundred counts above the dark phantom to ensure good SNR.

Throughout the ex vivo porcine desiccation experiment the spectrometer exposure time was adjusted for each measurement to ensure sufficient signal intensity count without saturating the detector. A dark phantom (fabricated with a high nigrosine concentration as an absorbing agent) was also measured using exposure time similar to those used for sample measurements. This allowed us to confirm that the sample measurements had intensity counts well above the dark phantom (dark noise) at all wavelengths. Looking at one of the lowest intensity periods 1200 nm, Baseline measurements: sample M had 1782 counts higher than dark phantom; sample M+A had 765 counts higher than dark phantom at the same integration times. At hour 8 of desiccation, sample M had 1594 counts higher than dark phantom; sample M+A had 1159 counts higher than dark phantom at the same integration times. All samples had counts several hundred counts above dark, indicating measurements were above the noise floor for all spectrometer measurements.
